# Development and Validation of A Fast, Simple And Specific Stability Indicating RP-HPLC Method for Determination of Dexpanthenol in Eye Gel Formulation

**DOI:** 10.22037/ijpr.2019.1100681

**Published:** 2019

**Authors:** Arash Mahboubi, Marjan Gholamreza Alviri, Minoo Afshar, Mahdieh Farhangi

**Affiliations:** a *Food Safety Research Center, School of Pharmacy, Shahid Beheshti University of Medical Sciences, Tehran, Iran.*; b *Department of Pharmaceutics, School of Pharmacy, Shahid Beheshti University of Medical Sciences, Tehran, Iran. *; c *Department of Pharmaceutics, Faculty of Pharmacy and Pharmaceutical Sciences, Tehran Medical Sciences, Islamic Azad University, Tehran 193956466, Iran. *; d *Department of Pharmaceutics, Faculty of Pharmacy, Hormozgan University of Medical Sciences, Bandar Abbas, Iran.*

**Keywords:** Dexpanthenol, Artificial tear, RP-HPLC, Validation, Carbomer

## Abstract

In this study a simple and reliable stability-indicating RP-HPLC method was developed and validated for analysis of Dexpanthenol in an artificial tear formulation. The chromatographic separation was performed on a HPLC C_18_ column (25.0 cm ´4.6 mm, 5 mm) using a mixture of 0.037 M monobasic potassium phosphate in water (adjusted with 0.1% (v/v) phosphoric acid to a pH of 3.2) and methanol (90:10). The flow rate was set at 1.5 mL/min and Dexpanthenol concentration was determined at λ_max _= 205 nm. The HPLC analysis method was validated in terms of linearity, precision, accuracy, specificity, and sensitivity according to International Conference on Harmonization (ICH) guidelines. The results indicated that the retention time was 8 min and no interferences were observed from the formulation excipients and stress degradation products. Linear regression analysis of data for the calibration plot showed a linear relationship between peak area and concentration over the range of 10 - 100 μg/mL; the regression coefficient was 0.996 and the linear regression equation was y = 20.011 x + 146.83. This HPLC method was precise and accurate in the range of 10 – 100 μg/mL. Also, the dexpanthenol concentration in artificial tear formulation was determined by this HPLC method, which was in accordance with the label claimed. This validated HPLC method could be used for routine analysis, quality control and the stability of analysis of eye gel containing dexpanthenol formulations.

## Introduction

**Figure 1:**
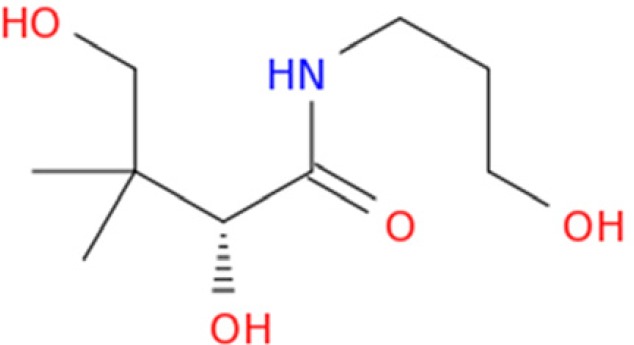
The chemical structure of Dexpanthenol

Dexpanthenol (Provitamin B_5_) is the alcohol analog of the panthetonic acid (Figure 1) which introduced by Roger J. Williams. This water-soluble vitamin showed higher biological activity and better stability in comparison with the acidic form. There is no report about toxic effect followed by oral or parenteral administration of pantothenic acid or any of its salts. Dexpanthenol is used in various multivitamins, skin and hair care preparations and cosmetic formulations due to its regenerating and anti-inflammatory properties (1-3). Dexpanthenol is yellow, viscous, hygroscopic liquid which is stable in neutral solutions but decomposes rapidly in acid or alkaline solution.

The dexpanthenol chemical structure has few UV chromophore groups and so its absorbance at high wavelength is low (1). By considering dexpanthenol chemical structure, different analytical methods such as colorimetric and fluorimetric methods, differential pulse voltammetry and HPLC / electrospray ionization-mass spectrometry have been developed (2-5). The USP analytical method for assaying dexpanthenol in bulk and formulations, is the non-aqueous titrimetric method (6).

Nowadays, chromatographic methods, including RP-HPLC are widely accepted and routinely used in pharmaceutical industry (7, 8). 

There are some studies which have reported RP-HPLC methods with UV detection for detection and quantification of dexpanthenol in pharmaceutical preparations. These analytical methods suffer from defects such as time-consuming analysis, or application of HPLC columns such as C_4 _(1, 9, 10).

The aim of the present study was developing a simple, fast, economical and ecofriendly RP-HPLC analysis method for dexpanthenol in artificial tear gel formulation. The stability of this analytical method for assaying dexpanthenol was also evaluated and it was indicated that the method could be used in a wide range of concentrations. 

## Experimental

Dexpanthenol USP Reference Standard were obtained from Sigma (St. Louis, MO). The placebo and dexpanthenol containing artificial tear gel (30 mg/mL) formulations were obtained from Nosha Pharmed (Tehran, Iran). The HPLC-grade methanol, phosphoric acid, and monobasic potassium phosphate salt were purchased from Merck (Darmstadt, Germany).

*Preparation of placebo artificial tear formulation*

The placebo formulation was prepared by dissolving similar component of the formulation including carbomer 934, EDTA, and benzalkonium chloride in deionized water. Triethanolamine (TEA) was added to the solution for gel preparation. The formulation pH was adjusted to7.7 – 7.9. 

**Figure 2:**
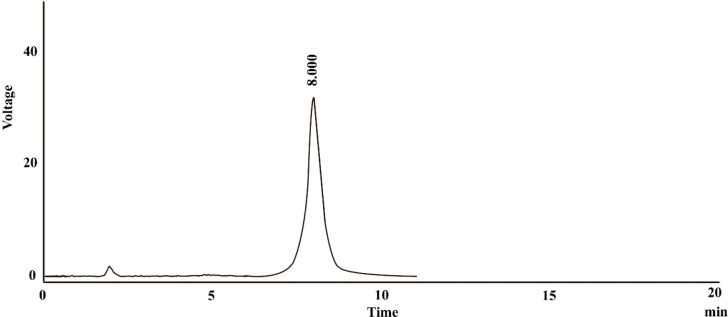
The dexpanthenol chromatogram

**Figure 4:**
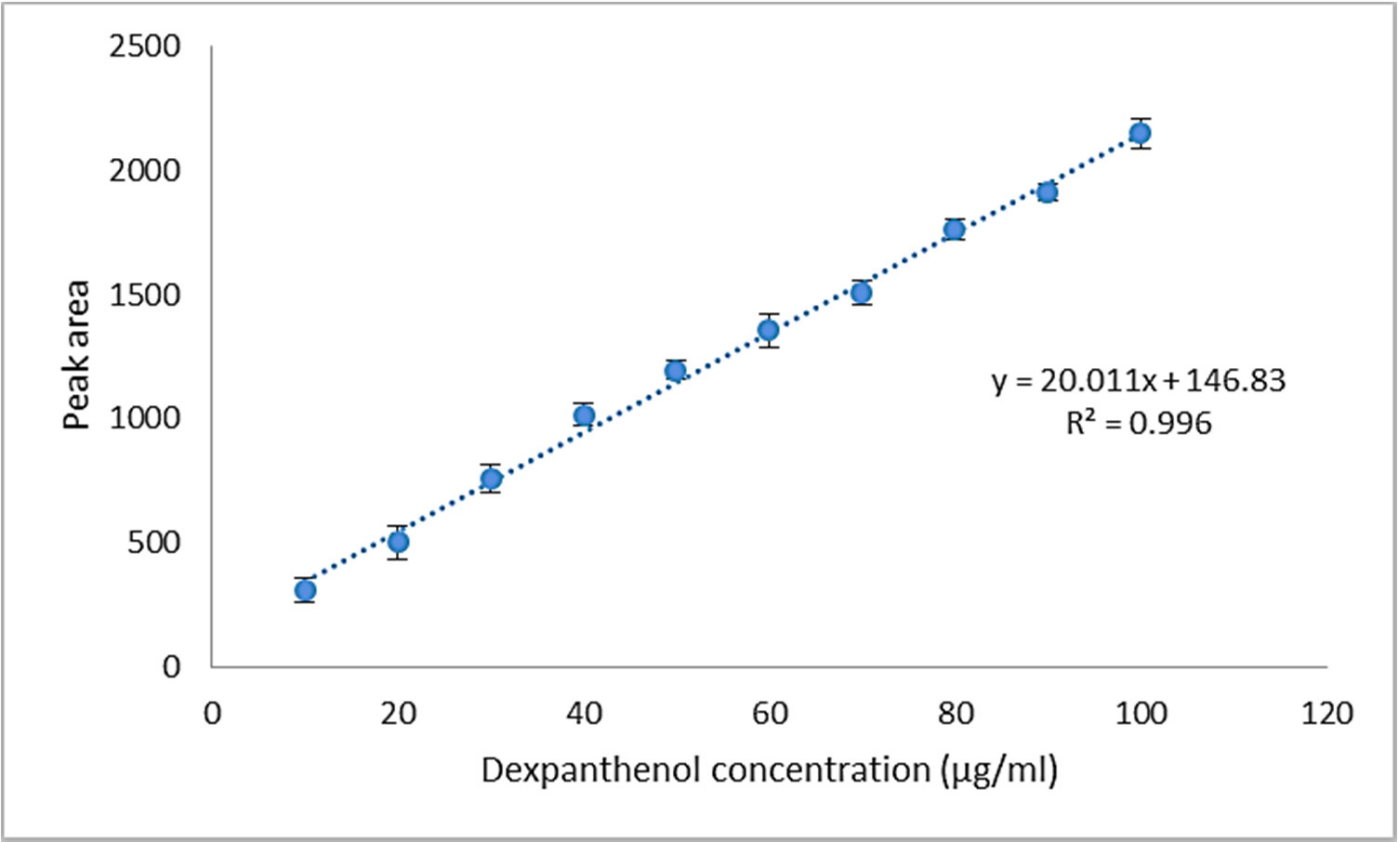
Linearity plot for dexpanthenol drug substance

**Table 1:** Results of the forced degradation of dexpanthenol (n = 3)

**Stress conditions**	**Degradation (%)**	**CV (%)**
0.1 M HCl, 1 h	No degradation	-
0.1 M HCl, 2 h	No degradation	-
0.1 M HCl, 6 h	0.62	4.11
0.1 M NaOH, 1 h	20.8	3.75
0.1 M NaOH, 2 h	35.5	7.21
0.1 M NaOH, 6 h	75.8	2.64
3 %V/V H2O2, 1 h	No degradation	-
UV light, 1 h	0.54	6.97
40 °C, 1 h	No degradation	-
40 °C, 2 h	No degradation	-
40 °C, 6 h	0.97	2.81

**Table 2:** Results of the accuracy of the developed method (n = 3)

**Sample concentration (µg/mL)**	**Recovery (%)**	**CV (%)**
30	99.67	0.87
70	98.59	1.05
90	100.90	0.75

**Table 3:** Intra-and inter-day precisions of the HPLC method for determination of dexpanthenol (n = 3)

**Sample concentration (µg/mL)**	**Intra-day precision**		**Inter-day precision**	
	**Mean ± SD**	**CV (%)**	**Mean ± SD**	**CV (%)**
20	23.20 ± 0.28	0.71	24.62 ± 0.41	1.02
60	65.41 ± 0.47	1.05	63.94 ± 0.53	1.19
80	84.07 ± 0.42	0.85	86.12 ± 0.48	0.99

*HPLC instrumentation*

In this study, the HPLC system (Knauer, Germany) consisted of a Wellchrom K-1001 pump and a Wellchrom K-2600 UV detector was used. The HPLC column was a MZ analytical C_18_ (25.0 cm × 4.6 mm, 5 µm, Germany). The sample injector was KNAUER manual injector. Integration package EZ Chrom Elite (version 3.1.7) software was used for data analysis. 

*Chromatographic conditions and mobile phase preparation*

The test was performed, under ambient and isocratic conditions. The HPLC system was equipped with C_18_ column (25.0 cm × 4.6 mm, 5 µm). The mobile phase was a mixture of 0.037 M monobasic potassium phosphate in water (adjusted with 0.1% (v/v) phosphoric acid to a pH of 3.2) and methanol (90:10); filtrated by a 0.45 µm membrane filter and degassed before use. The flow rate was set at 1.5 mL/min. The sample was monitored at λ_max_ = 205 nm. The injection volume was 50 µL. 

*Method validation*

Validation of the proposed method was carried out according to the ICH guidelines. It includes specificity, precision, linearity, accuracy, as well as determining limit of detection (LOD) and limit of quantification (LOQ) (11).

*Specificity*

The specificity of the proposed method was assessed by checking any possible interference effect of the formulation component. Moreover, the possible interferences of the by-products of the forced of dexpanthenol degradation were investigated. 

The specificity of the method was carried out by dissolving 5 mg of dexpanthenol and 5 mg of carbomer in 100 mL of deionized water, and the sample was analyzed for the dexpanthenol recovery.

According to the ICH guideline, dexpanthenol at the concentration of 1 mg/mLwas spiked to the placebo. This solution was evaluated for stability in Acid hydrolysis, Alkaline hydrolysis, Oxidative degradation, Thermal degradation, and Photostability.

Each stressed test solution was diluted with mobile phase and injected to the HPLC system (12). The stress conditions are described as follows.

*A: Acid hydrolysis*

The acid hydrolysis was carried out by adding 1 mL of 0.1 M HCl to 1 mL of test solution. The mixture was stirred (Labinco, The Netherlands) (600 rpm) at the room temperature and the samples were taken at 1, 2, and 6 h time intervals; the samples were collected and after neutralizing with 0.1 M NaOH solution, they were analyzed (7).

*B: Alkaline hydrolysis*

Briefly, 1 mL of the test solution was mixed with 1 mL of 0.1 M NaOH and stirred (Labinco, The Netherlands) (600 rpm) at room temperature. At predetermined time intervals (1, 2 and 6 h), the samples were collected and neutralized by adding 0.1 M HCl solution and they were analyzed (7).

*C: Oxidative degradation*

The test was carried out by adding 1 mL of 3 %V/V H_2_O_2_ to the test solution and stirring of the mixture at the room temperature in a dark place for 1 h and analyzed (13, 14).

*D: Thermal degradation*

The test solution was heated up to 40 °C for 6 h; the samples were collected at 1, 2, and 6 time intervals, cooled, and then analyzed.

*E: Photostability*

The test solution was exposed to UV light (254 nm) in a UV light chamber for 1 h and then analyzed (14-16). 

*Linearity*

In this test, the dexpanthenol quantitative determination range was 10 - 100 µg/mL. The stoke solution was prepared by spiking dexpanthenol to the placebo formulation. The working standard solutions were prepared by serial dilutions of the stock solution (100 µg/mL dexpanthenol) in the mobile phase. The calibration curve was constructed by plotting the peak area of dexpanthenol versus concentration of dexpanthenol. Every concentration levels were analyzed three times.

*Accuracy*

The accuracy of an analytical procedure expresses as the closeness of agreement between the experimental concentration and theoretical concentration. The accuracy of the HPLC method was established by injecting three concentrations of calibration curve (30, 70 and 90 µg/mL). The solutions were injected three times into HPLC and the mean peak area was obtained. The recovery was calculated using the following equation:

Recovery (%) = $\frac{\mathrm{Experimental concentration}}{\mathrm{Theoretical concentration}}$ × 100 (13)                     Equ. 1

*Precision*

The intra-day (repeatability) and inter-day (reproducibility) precision was evaluated by assaying three replicates of standard solutions at three different concentrations in the range of calibration curve (20, 60 and 80 µg/ml) in the same day and in 3 different days, respectively. The coefficient of variation (CV%) of peak areas was calculated. 

*Sensitivity*

The LOQ was established during the evaluation of the linear range of calibration curve and at a signal to noise ratio (S/N) of 10. The LOD was established at a signal to noise ratio (S/N) of 3.3. 

*Dexpanthenol assay in eye gel formulation*

For assaying dexpanthenol in the product, 1 mL of dexpanthenol containing formulation (30 mg/mL) was transferred to a 200 mL volumetric flask and diluted with deionized water. Then 1 mL of this solution was diluted with mobile phase in 1: 3 ratio and analyzed after filtering through a 0.45 µm membrane filter. The procedure was repeated 5 times and drug content was quantified using the standard calibration curve. 

## Results and Discussion

*Method development*

Assaying active pharmaceutical ingredients in bulk and dosage forms is essential for ensuring the quality of the pharmaceutical products. The procedure should be done by appropriate methods which must be simple, fast, and reliable, as well as cost-effective (7). In the present study, a RP-HPLC method for assaying dexpanthenol in artificial tear formulation was developed. As is presented in Figure 2, the dexpanthenol chromatogram showed a sharp peak at 8 min which could be considered as a proper retention time. 

*Method validation*

*Specificity*

The specificity of the developed method was determined by injecting a mixture of dexpanthenol and formulation excipients under normal and stress conditions. Table 1 shows the results of the forced degradation of dexpanthenol. The results declared that dexpanthenol was stable under acidic conditions and just 0.62 % of drug was degraded after 6 h. The results reflected the oxidative stability of the drug. Dexpanthenol also showed good stability in thermal and photo degradation by slightly decomposition, i.e. 0.97% and 0.54%, respectively. Dexpanthenol was hydrolyzed under alkaline conditions in a time-dependent manner. As Figure 3 shows, the dexpanthenol retention time did not change and the stability indicating capability of the method was established by the separation of dexpanthenol peak from the degraded samples and excipients (7, 13, 15).

Figure 3. Representative chromatograms of A) standard dexpanthenol solution, B) standard dexpanthenol and carbomer mixture, C) 0.1 M HCl, 6 h, D) 0.1 M NaOH, 6 h, E) 3 %V/V H_2_O_2 _, 1 h, F) UV light, 1 h, G) 40 °C, 6 h.

*Linearity*

Figure 4 shows the plot of peak area responses against the concentration of dexpanthenol. The plot is linear over the concentration range of 10 - 100 μg/mL yielding a regression equation y = 20.011 x + 146.83 with a coefficient of correlation equal to 0.996.

*Accuracy*

Table 2 shows the results of accuracy assessment obtained for different dexpanthenol concentrations. The results indicated the percent recoveries ranging from 98.59 to 100.90% with CVs ranging from 0.75 to 1.05% which comply with the acceptance criteria proposed (% Recovery range: 80- 120%) (6).

*Precision*

The precision of the method was determined by repeatability (intra-day precision) and intermediate precision (inter-day precision) of the dexpanthenol standard solutions (Table 3). The CV% range was obtained as 0.71 – 1.05 and 0.99 – 1.19 for intra-day and inter-day precision studies, respectively.

*Sensitivity*

Based on the results, the LOD and LOQ values for dexpanthenol by this HPLC method are 3 μg/mL and 8.5 μg/mL, respectively.

*Dexpanthenol assay in eye gel formulation*

The developed HPLC method was applied for the assessment of dexpanthenol in a commercial artificial tear formulation. The results of the fifth analysis indicated that the amount of dexpanthenol was 28.42 ± 0.13 mg/mL (dexpanthenol recovery = 94.7%) of the formulation which was in accordance with the label claimed.

## Conclusion

The aim of the present study was to develop a HPLC method for analysis of dexpanthenol in artificial tear gel formulations. The developed HPLC method is an easy and stability-indicating method. The results indicated that this method was proved to be specific, accurate, precise, and reproducible. Time-saving is a valuable factor in the development of this analytical method. As the results indicated, the total analysis time was less than 10 min while the use of organic solvent (methanol) was low and the routinely used C_18_ column was applied; so the use of this analytical method in pharmaceutical industry, in comparison to other methods, would be notable. The method was successfully used, without interference from the degradation products and /or excipients. Finally, the improved method was successfully performed to the analysis of dexpanthenol in eye gel and thus, it can be used for routine analysis and quality control of eye gel containing dexpanthenol formulations. 

## References

[1] Kulikov AU, Zinchenko AA (2007). Development and validation of reversed phase high performance liquid chromatography method for determination of dexpanthenol in pharmaceutical formulations. J. Pharm. Biomed. Anal.

[2] Shehata MAM, Tawakkol S, Abdel Fattah LE (2002). Colorimetric and fluorimetric methods for determination of panthenol in cosmetic and pharmaceutical formulation. J. Pharm. Biomed. Anal.

[3] Chen Z, Chen B, Yao S (2006). High-performance liquid chromatography/electrospray ionization-mass spectrometry for simultaneous determination of taurine and 10 water-soluble vitamins in multivitamin tablets. Anal. Chim. Acta.

[4] Lai-Hao W, Shih-Wen T (2001). Direct determination of d-panthenol and salt of pantothenic acid in cosmetic and pharmaceutical preparations by differential pulse voltammetry. Anal. Chim. Acta.

[5] Havl´ıkov´a L, Matysov´a L, Nov´akov´a L, Solich P (2006). HPLC determination of calcium pantothenate and two preservatives in topical cream. J Pharm Biomed Anal.

[6] USP ( 2006). USP 40 –NF 35, Validation of Compendial Procedures <1225>.

[7] Darsazan B, Shafaati A, Mortazavi SA, Zarghi A (2017). A Simple and Specific Stability-Indicating RP-HPLC Method for Routine Assay of Adefovir Dipivoxil in Bulk and Tablet Dosage Form. Iran. J. Pharm. Res.

[8] Shokraneh F, Asgharian R, Abdollahpour A, Ramin M, Montaseri A, Mahboubi A (2015). A Novel High Performance Liquid Chromatographic Method for Determination of Nystatin in Pharmaceutical Formulations by Box–Behnken Statistical Experiment Design. Iran. J. Pharm. Res.

[9] Stojanka Vc, Biljana Sc, Jelena Vc, Ljiljana Pzc-Ac, Goran Rc, Dragan Mc (2008). Simultaneous determination of some water-soluble vitamins and preservatives in multivitamin syrup by validated stability-indicating high-performance liquid chromatography method. J. Chromatogr. A.

[10] Heudi O, Kilinc T, Fontannaz P (2005). Separation of water-soluble vitamins by reversed-phase high performance liquid chromatography with ultra-violet detection: Application to polyvitaminated premixes. J. Chromatogr. A.

[11] (2005). ICH Validation of analytical procedures: text and methodology Q2 (R1).

[12] (2003). ICH Stability Testing of New Drug Substances and Products Q1A (R2).

[13] Serri A, Moghimi HR, Mahboubi A, Zarghi A (2017). Stability-indicating HPLC method for determination of vancomycin hydrochloride in the pharmaceutical dosage forms. Acta Pol. Pharm.

[14] Khoshkam R, Afshar M (2014). Validation of a Stability-Indicating RP-HPLC Method for Determination of l-Carnitine in Tablets. Int. Sch. Res. Notices.

[15] Annapurna MM, Mohapatro C, Narendra A (2012). Stability-indicating liquid chromatographic method for the determination of Letrozole in pharmaceutical formulations. JPA.

[16] Sadeghi F, Navidpour L, Bayat S, Afshar M (2013). Validation and Uncertainty Estimation of an Ecofriendly and Stability-Indicating HPLC Method for Determination of Diltiazem in Pharmaceutical Preparations. J. Anal. Methods Chem.

